# Efficacy of Insulin Eye Drops in the Treatment of Corneal Ulcers in Patients with Facial Nerve Palsy and Lagophthalmos: A Retrospective Case–Control Study

**DOI:** 10.3390/medicina61111991

**Published:** 2025-11-06

**Authors:** Marija Jelić Vuković, Suzana Matić, Dubravka Biuk, Miro Kalauz, Andrijana Kopić, Vedran Nemet, Ivana Strunje, Ivanka Maduna, Stipe Vidović, Ena Kolak

**Affiliations:** 1 Department of Ophthalmology, University Hospital Centre Osijek, 31000 Osijek, Croatia; marija.jelic.vukovic@gmail.com (M.J.V.); suzimatic72@gmail.com (S.M.); dubravka.biuk@gmail.com (D.B.); andrijanakopic@gmail.com (A.K.); nemet.vedran@gmail.com (V.N.); ipivic.kovacevic@gmail.com (I.S.); ivankamadun@gmail.com (I.M.); stipevidovic1@gmail.com (S.V.); 2 Faculty of Medicine, Josip Juraj Strossmayer University of Osijek, 31000 Osijek, Croatia; 3 Department of Ophthalmology, University Hospital Centre Zagreb, 10000 Zagreb, Croatia; miro.kalauz@gmail.com; 4 School of Medicine, University of Zagreb, 10000 Zagreb, Croatia; 5 Primary Health Care Center, Osijek-Baranja County, 31000 Osijek, Croatia

**Keywords:** administration, topical, cornea, corneal diseases, corneal ulcer, epithelium, corneal, facial paralysis, insulin, lagophthalmos, randomized controlled trial, re-epithelialization treatment outcome

## Abstract

*Background and Objectives:* Corneal ulcers in patients with lagophthalmos due to facial nerve palsy pose a significant therapeutic challenge. Topical insulin has emerged as a promising adjuvant therapy for enhancing corneal re-epithelialization. The aim of this study is to evaluate the efficacy of insulin drops compared with lipid-based artificial tears, and to compare the corneal healing rate in achieving complete epithelialization within 30 days in diabetic patients. *Materials and Methods:* This retrospective case–control study included 32 patients with facial nerve palsy and lagophthalmos who developed an exposed corneal ulcer, of whom 20 received topical insulin and 12 received lipid-based artificial tears. The primary outcome was complete epithelialization at day 30. Additional variables included age, sex, and baseline defect characteristics. *Results:* By day 30, complete epithelialization was achieved in 18 of 20 patients (90%) in the insulin group compared with 5 of 12 (41.7%) in the control group. Binary logistic regression analysis confirmed significantly higher odds of healing with insulin treatment (OR = 10.8; 95% CI 1.8–63.9). No systemic adverse events or signs of hypoglycemia were observed. *Conclusions:* Topical insulin significantly accelerates corneal epithelialization in patients with facial nerve palsy and exposed ulcers, offering safe and effective adjuvant therapy for a high-risk population. Despite promising results, the study’s limitations—including small sample size, single-center design, and retrospective nature—highlight the need for larger, multicenter prospective studies to confirm efficacy, optimize dosing, and further evaluate long-term safety.

## 1. Introduction

Corneal ulcer is defined as a defect of the corneal epithelium with underlying stromal involvement and represents a condition that can severely compromise vision [1]. It is typically associated with tissue excavation, infiltration, and necrosis. Even with timely treatment, serious complications can develop, including corneal scarring, thinning or perforation, secondary glaucoma, cataract, anterior and posterior synechiae, and permanent loss of vision. A variety of predisposing factors have been identified. Local ocular risk factors include trauma, dry eye syndrome, vitamin A deficiency, chronic dacryocystitis, ectropion or entropion, trichiasis, distichiasis, exophthalmos, lagophthalmos, contact lens wear, and irregular or prolonged corticosteroid use. Systemic risk factors, such as malnutrition, diabetes mellitus, chronic alcoholism, drug abuse, malignancy, acquired immunodeficiency syndrome, and immunosuppression, further increase the susceptibility to corneal ulcer development [2,3,4,5].

Lagophthalmos refers to the inability to achieve complete eyelid closure [6]. Normal eyelid closure with an intact blink reflex is essential for maintaining tear film stability and a healthy ocular surface. When this mechanism is impaired, patients are predisposed to corneal exposure, accelerated tear film evaporation, and subsequent exposure keratopathy. If persistent, this condition may progress to chronic epithelial defects and corneal ulceration. Among many etiologies of lagophthalmos, the primary cause is facial nerve palsy, resulting in paralytic lagophthalmos [7]. In a retrospective analysis of 1870 patients with facial nerve paralysis, lagophthalmos was present in 49% of eyes with ocular surface exposure, and 12.9% of these eyes developed corneal ulcers, with 0.6% progressing to perforation [8].

The annual incidence of corneal ulcers in the United States is estimated to be between 30,000 and 75,000 [9]. In Europe, precise standardized data is lacking. The treatment of chronic corneal ulcers is complex and non-standardized, largely depending on the severity of the disease, and as such still represents a significant challenge in ophthalmology. Early chronic ulcers are often difficult to distinguish from dry eye syndrome and are typically treated with lubricating eye drops or gels that are intended to promote healing and preserve ocular surface integrity. When such therapy proves insufficient, more advanced interventions are required. Common treatments for moderate to severe chronic ulcers include the use of serum eye drops (SED), possibly in combination with contact lenses, amniotic membrane transplantation (AMT), or emergency corneal transplantation (KT) [10,11,12].

Insulin therapy has traditionally been associated with glycemic control, but recent studies suggest that insulin may also play a significant role in corneal epithelial regeneration [13,14,15,16]. Multiple in vitro and preclinical studies have demonstrated that insulin promotes proliferation, migration, and desquamation of corneal epithelial cells (CECs), while modulating inflammatory processes essential for corneal ulcer healing and maintaining corneal transparency. In vitro studies have shown that insulin promotes cell survival and inhibits apoptosis, thereby accelerating re-epithelialization of CECs through activation of the PI3K and ERK1/2 signaling pathways, compared with leptin, which has no such effect [13,14]. Some studies also indicate the association of insulin with activation of the Wnt/β-catenin signaling pathway, which may contribute to corneal nerve regeneration [15]. In vivo studies, particularly in streptozotocin-induced diabetic keratopathy in rats, have shown that topical insulin (e.g., 1 U/mL, 4× daily) significantly accelerates corneal healing and restores corneal sensation to a level comparable to healthy controls [16]. A retrospective analysis of six patients with neurotrophic corneal ulcers who had failed to respond to standard medical and surgical treatments demonstrated that topical insulin may be a safe and effective therapy. Patients received insulin drops, and complete corneal re-epithelialization was achieved within 7 to 25 days, with no reported systemic side effects [17]. Importantly, topical insulin administration provides local benefits without systemic risk of hypoglycemia, making it a promising safe and effective therapeutic strategy for patients with chronic or neurotrophic corneal ulcers. Despite the growing interest in the use of topical insulin in ophthalmology, available clinical evidence remains limited, especially in patients with facial nerve palsy and lagophthalmos, a group in which standard therapies are often insufficient. The aim of this retrospective case–control study was to evaluate the efficacy of insulin drops compared with lipid-based artificial tears, with particular emphasis on corneal healing rate.

## 2. Materials and Methods

This retrospective case–control study was conducted at the Eye Clinic University Hospital Osijek between 1 January 2025, and 20 September 2025, and included 32 adult patients with facial nerve palsy and lagophthalmos who developed an exposed corneal ulcer and had a diagnosis of diabetes mellitus. The study was conducted in accordance with the principles of the Declaration of Helsinki and was approved by the Ethics Committee of the University Hospital Centre Osijek (protocol code R1-10457/2025; date of approval: 22 September 2025).

Patients were classified as cases if they received topical insulin eye drops (n = 20), and as controls if they were treated with artificial tears containing a lipid component (n = 12). Exclusion criteria included active herpetic keratitis, systemic diseases directly affecting the cornea, and intracorneal procedures within the previous three months, known allergy or hypersensitivity to insulin or components of artificial tears. Treatment information was extracted from medical records and included topical insulin drops (1 IU/mL Humulin R (Eli Lilly and Company, Indianapolis, IN, USA) in polyethylene glycol/polypropylene glycol (PEG/PPG), administered four times daily for 30 days) or artificial tears with a lipid component (Systane^®^ Complete (Alcon Laboratories, Fort Worth, TX, USA)), commonly used for dry eye management, alongside standard of care (SOC) measures such as antibiotic regimen in cases of secondary infection and identical physical protection using a moist shell [18]. The Systane^®^ Complete formulation contains a lipid emulsion and the preservative POLYQUAD^®^ 0.001% (Alcon Laboratories, Fort Worth, TX, USA) [19].

Data obtained from patient records included fluorescein staining according to Oxford staining grade, tear break-up time (TBUT), Schirmer I test results, visual acuity expressed in logarithm of the minimum angle of resolution (logMAR) units, and subjective symptoms assessed by the Ocular Surface Disease Index (OSDI) questionnaire [20,21,22]. The primary outcome was complete corneal epithelialization within 30 days. Additional variables such as age, sex, and baseline defect characteristics were also collected.

### Statistical Analysis

Descriptive statistics were used to summarize baseline characteristics. Differences between the two groups were assessed using the chi-square test, Fisher’s exact test, or the Fisher–Freeman–Halton exact test for categorical variables, and *t*-tests or Mann–Whitney tests for continuous variables, as appropriate. The association between treatment (topical insulin versus lipid-containing artificial tears) and the primary outcome of complete corneal epithelialization at 30 days was evaluated using binary logistic regression, with calculation of odds ratios (OR) and 95% confidence intervals (CI). A Kaplan–Meier analysis was used to illustrate the dynamics of corneal healing over time. Differences in categorical variables between the insulin and control groups were assessed using the chi-square test. The Wilcoxon signed-rank test and the Mann–Whitney U test were performed to assess the significance of changes in logMAR values within and between groups, respectively. Statistical significance was set at α = 0.05 (two-tailed). All analyses were performed using IBM SPSS Statistics (version 28; IBM Corp., Armonk, NY, USA). The study report was prepared in accordance with current guidelines for reporting research in biomedicine and health sciences [23,24].

## 3. Results

A total of 32 patients aged 40 to 70 years were included in the study. At baseline, no significant differences were observed between the insulin and control groups regarding age, sex, Oxford staining score, Schirmer I test, TBUT, or logMAR visual acuity (all *p* > 0.05). Baseline characteristics of the study participants are shown in Table 1.

**Figure 1:**
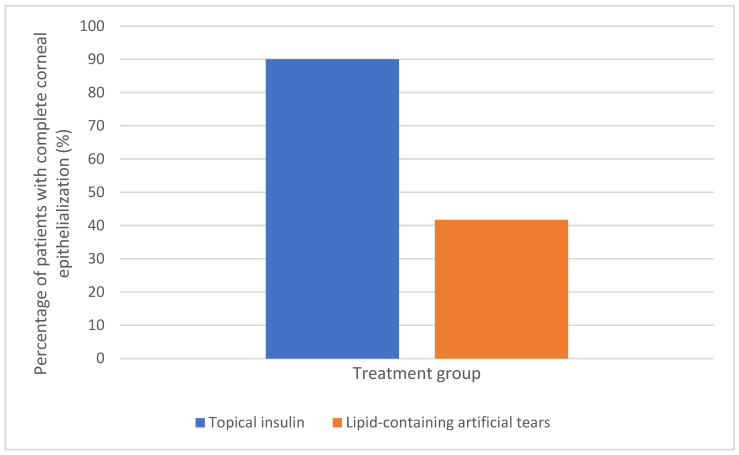
Proportion of patients achieving complete corneal epithelialization at day 30 in insulin and control groups.

**Figure 2:**
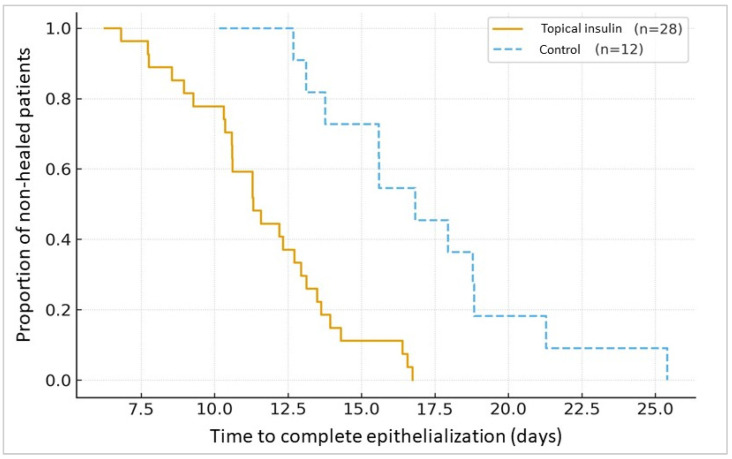
Kaplan–Meier analysis of time to complete corneal epithelialization.

By day 30, complete corneal epithelialization was achieved in 18 out of 20 patients treated with topical insulin drops (90%), compared with 5 of 12 patients in the control group (41.7%). Corneal healing outcomes by day 30 are presented in Figure 1. This difference was statistically significant (*p* = 0.012). To further illustrate the dynamics of corneal healing over time, a Kaplan–Meier analysis was performed, which is presented in Figure 2. Binary logistic regression analysis showed that the odds of complete epithelialization were significantly higher in the insulin group, with an odds ratio of 10.8 (95% CI 1.8–63.9). No systemic adverse events or signs of hypoglycemia were observed in any patient. At baseline, no significant differences were observed between the groups regarding ulcer area, defect depth, or duration prior to treatment initiation (all *p* > 0.05), indicating comparable initial clinical severity. After 30 days of treatment, a significant improvement in LogMAR visual acuity was observed in the insulin group, whereas the improvement in the control group did not reach statistical significance; secondary outcomes are also summarized in Table 2.

## 4. Discussion

In this retrospective case–control study, topical application of insulin drops (1 U/mL) was associated with a significantly higher likelihood of complete corneal epithelialization in patients with facial nerve palsy and lagophthalmos compared to artificial tears with a lipid component. By day 30, complete epithelialization was observed in 90% of patients treated with topical insulin versus 41.7% in the control group, corresponding to a markedly increased odds of healing (OR = 10.8; 95% CI 1.8–63.9). However, the relatively wide confidence interval (CI) points to the limited sample size, emphasizing the necessity for larger studies to validate these outcomes. No systemic adverse effects or signs of hypoglycemia were observed.

These findings align with preclinical evidence showing the proliferative and pro-reparative effect of insulin on the corneal epithelium and are supported by prior clinical research. The systematic review by Wouters et al. included literature searches in Medline, Embase, and Web of Science, along with additional manual searches using relevant keywords, covering all articles published from January 2005 to January 2024. Sixteen studies were reviewed, including two randomized controlled trials. Insulin has proven to be a promising and effective adjuvant treatment for neurotrophic keratopathy, aiding the healing of epithelial defects while offering many advantages such as broad availability and low cost [25].

Similarly, Soares et al. reported high rates of complete epithelialization (75–90%) without significant side effects, with therapy durations ranging from 7 days to 8 weeks, which aligns closely with the results of our study [26]. In a randomized controlled trial by Dasrilsyah et al. involving diabetic patients with postoperative corneal epithelial defects, topical insulin (0.5 units/drop, 4 times per day) was again shown to be superior to artificial tears (Vismed, sodium hyaluronate 0.18%, 4 times per day) regarding healing speed (*p* = 0.010 to 0.009) and was well tolerated [27]. These findings of accelerated epithelialization in the high-risk group are consistent with our results.

A systematic review by Krolo et al. supports these observations by identifying two additional studies demonstrating that topical insulin administration provides better results in the treatment of postoperative corneal epithelial defects in patients with diabetes [28,29,30]. The review included 17 articles and identified a total of 387 eyes treated with topical insulin for various ocular surface pathologies, with more than half of the patients (61%) having concomitant diabetes mellitus.

On the other hand, the study by Abdi et al., which assessed the clinical efficacy of topical insulin eye drops in patients with refractory persistent epithelial defects (PED), showed somewhat lower success rates (approximately 70%), which was heavily reliant on the initial defect size. This prospective non-randomized study included 23 patients unresponsive to conventional therapy and administered insulin eye drops (1 U/mL) four times daily. Primary outcomes were the rate of resolution of epithelial defects and the time to complete corneal re-epithelialization. Results indicated that 16 patients (69.6%) achieved improvement during follow-up (maximum 50 days). Insulin eye drops significantly reduced the area of corneal injury in 75% of patients with small epithelial defects (5.5 mm^2^ or less) within 20 days. Only 61% of patients with moderate epithelial defects (5.51–16 mm^2^) showed significant recovery within 20–30 days. Additionally, 71% of patients with defect sizes over 16 mm^2^ exhibited significant improvement in corneal epithelial wound healing within approximately 50 days. Overall, topical insulin accelerated wound healing and reduced the PED area [15].

In comparison, our retrospective case–control study in patients with facial nerve palsy and severe lagophthalmos—a particularly high-risk cohort—achieved a higher rate of complete epithelialization in shorter period. This difference likely reflects the characteristics of our study population and documented adherence to the prescribed treatment in medical records. For the few ulcers that did not achieve complete epithelialization, alternative strategies are commonly considered in clinical practice. These include autologous serum eye drops and amniotic membrane transplantation, which can provide additional support for epithelial healing in refractory cases [10,11,12]. Other supportive measures, such as tarsorrhaphy to protect the cornea, bandage contact lenses, and the use of growth factor or anti-inflammatory drops, may also be employed depending on the extent of corneal exposure and epithelial defect [31,32,33,34]. Although some patients in the cohort had concomitant diabetes mellitus, glycemic parameters such as HbA1c or fasting glucose were not systematically collected, since diabetes was not an inclusion criterion. This limits our ability to explore potential associations between systemic glycemic control and corneal healing response. However, as our study focused on patients with facial nerve palsy and severe lagophthalmos, ulcer formation was primarily exposure-related rather than metabolic in origin. Future studies with detailed metabolic profiling of diabetic subpopulations could further elucidate insulin’s reparative potential in diabetic keratopathy. Nevertheless, certain limitations must be acknowledged. The relatively small sample size, single-center design, and retrospective nature limit the generalizability of the findings. Moreover, the study population consisted exclusively of patients with facial nerve palsy, which, although representing a high-risk group, may not fully reflect outcomes in patients with PED causes. Given the retrospective design, these analyses assess associations rather than causal effects. Future studies should therefore aim to confirm these findings in larger, multicenter cohorts, encompassing diverse patient populations and standardized treatment protocols. Furthermore, integrating systematic metabolic and inflammatory profiling, together with quantitative imaging-based ulcer measurements, would enhance understanding of insulin’s mechanism of action and help define which patient subsets may benefit most.

## 5. Conclusions

Our data confirm the efficacy of topical insulin reported in previous studies, while providing additional evidence from a retrospective case–control study in a unique, high-risk patient population. Nevertheless, due to study limitations—including the relatively small sample size and single-center design—larger prospective randomized trials are needed to further confirm the efficacy, determine the optimal dosing regimen, and comprehensively evaluate the safety profile. Future work should focus on standardized drop preparation, stability control, and multicenter study designs.

## Figures and Tables

**Table 1:** Baseline characteristics of the study participants.

	Insulin Group (n = 20)	Control Group (n = 12)	*p*-Value	Test
Female gender (n [%])	11 (55%)	7 (58%)	1.00	Continuity corrected chi-square test
Male gender (n [%])	9 (45%)	5 (42%)		
Age (years, median [IQR])	58 (52–65)	60 (50–64)	0.58	*t*-test
Oxford staining score	2 (2–3)	2 (2–3)	0.47	*t*-test
Schirmer I test (millimeters, median [IQR])	7 (5–9)	8 (5–10)	0.55	*t*-test
TBUT (median [IQR])	6 (5–7)	6 (5–8)	0.73	*t*-test
visual acuity (logMAR, median [IQR])	0.4 (0.3–0.5)	0.4 (0.3–0.6)	0.64	*t*-test
OSDI score	45 (40–55)	46 (42–56)	0.59	*t*-test
**Ulcer characteristics**				
Ulcer size (mm^2^), n (%)	Small (<2): 7 (35%) Medium (2–5): 9 (45%) Large (>5): 4 (20%)	Small (<2): 4 (33%) Medium (2–5): 6 (50%) Large (>5): 2 (17%)	1.00	Fisher–Freeman–Halton exact test
Defect depth, n (%)	Superficial: 13 (65%) Stromal: 7 (35%)	Superficial: 7 (58%) Stromal: 5 (42%)	0.72	Fisher’s exact test
Defect duration before therapy (days), median [IQR]	14 [10,11,12,13,14,15,16,17,18,19,20]	15 [11,12,13,14,15,16,17,18,19,20,21,22]	0.73	Mann–Whitney test

IQR, interquartile range; TBUT, tear break-up time; OSDI, Ocular Surface Disease Index; logMAR VA, logarithm of the minimum angle of resolution visual acuity. Categorical variables were analyzed using χ^2^ or Fisher’s exact test, and continuous variables using *t*-test or Mann–Whitney U test, according to normality of distribution.

**Table 2:** Secondary outcomes of the study participants.

**Outcome**	**Insulin Group (n = 20)**	**Control Group (n = 12)**	**Test**	***p*-Value**	**95% CI for Difference in Proportions**
Complete epithelialization	90%	41.7%	χ^2^ = 6.33	0.012	16–80%
TBUT ≥ 2 s improvement	75%	33%	χ^2^ = 4.56	0.033	6–72%
Schirmer I ≥ 3 mm improvement	60%	25%	χ^2^ = 3.92	0.048	2–65%
OSDI score reduction ≥ 20%	70%	33%	χ^2^ = 4.18	0.041	4–68%
Visual acuity ≥ 0.1 logMAR improvement	50%	25%	χ^2^ = 2.11	0.146	−10–61%
**Visual Acuity (logMAR)**					
**Parameter**	**Insulin Group (n = 20)**	***p*** **(Within-Group)**	**Control Group** **(n = 12)**	***p* (Within-Group)**	***p* (Between-Groups, day-30)**
Baseline logMAR (median [IQR])	0.40 [0.30–0.50]	-	0.40 [0.30–0.60]	-	-
Final logMAR − day-30 (median [IQR])	0.20 [0.10–0.30]	0.012 (Wilcoxon)	0.30 [0.20–0.50]	0.081 (Wilcoxon)	0.045 (Mann–Whitney)
Change (ΔlogMAR = day-30 − baseline)	−0.20 [−0.30 to −0.10]	-	−0.10 [−0.20 to −0.00)]	-	-

Data are presented as median [interquartile range]. Within-group comparisons were performed using the Wilcoxon signed-rank test, and between-group comparison of final values was performed using the Mann–Whitney U test.

## Data Availability

The data supporting the findings of this study are not publicly available due to ethical restrictions and the presence of personal information. Data can be made available from the authors upon reasonable request, ensuring that all data remain de-identified to protect patient privacy.

## Abbreviations

The following abbreviations are used in this manuscript:

AMT	Amniotic Membrane Transplantation
AS-OCT	Anterior Segment Optical Coherence Tomography
CECs	Corneal Epithelial Cells
OR	Odds Ratio
CI	Confidence Interval
IQR	Interquartile range
logMAR	Logarithm of the Minimum Angle of Resolution
PED	Persistent Epithelial Defect
PPG	Polypropylene Glycol
PEG	Polyethylene Glycol
SED	Serum Eye Drops
SOC	Standard of Care
TBUT	Tear Break-Up Time
OSDI	Ocular Surface Disease Index
KT	Corneal Transplantation
